# Novel therapeutics for hydrocephalus: Insights from animal models

**DOI:** 10.1111/cns.13695

**Published:** 2021-06-20

**Authors:** Chuansen Wang, Xiaoqiang Wang, Changwu Tan, Yuchang Wang, Zhi Tang, Zhiping Zhang, Jingping Liu, Gelei Xiao

**Affiliations:** ^1^ Department of Neurosurgery Xiangya Hospital Central South University Changsha Hunan China; ^2^ Diagnosis and Treatment Center for Hydrocephalus Xiangya Hospital Central South University Changsha Hunan China; ^3^ National Clinical Research Center for Geriatric Disorders Xiangya Hospital Central South University Changsha Hunan China; ^4^ Department of Pediatric Neurosurgery Xinhua Hospital Shanghai Jiaotong University School of Medicine Shanghai China; ^5^ Department of Neurosurgery Hunan Cancer Hospital and the Affiliated Cancer Hospital of Xiangya School of Medicine Central South University Changsha Hunan China

**Keywords:** animal models, cerebrospinal fluid, hydrocephalus, nonsurgical treatment, subarachnoid space

## Abstract

Hydrocephalus is a cerebrospinal fluid physiological disorder that causes ventricular dilation with normal or high intracranial pressure. The current regular treatment for hydrocephalus is cerebrospinal fluid shunting, which is frequently related to failure and complications. Meanwhile, considering that the current nonsurgical treatments of hydrocephalus can only relieve the symptoms but cannot eliminate this complication caused by primary brain injuries, the exploration of more effective therapies has become the focus for many researchers. In this article, the current research status and progress of nonsurgical treatment in animal models of hydrocephalus are reviewed to provide new orientations for animal research and clinical practice.

## Graphical abstract

Inflammation, fibrosis, iron overload, etc., play an important role in the development of hydrocephalus. By targeting these mechanisms, researchers have discovered a series of drugs that have therapeutic effects on hydrocephalus.

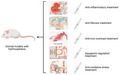

## 1 INTRODUCTION

Hydrocephalus is a disorder of the cerebrospinal fluid (CSF) physiology resulting in the expansion of cerebral ventricles with normal or high intracranial pressure.^1^ It is usually congenital or secondary to craniocerebral injury or intracranial infection. At present, surgical therapies are widely used to treat hydrocephalus, while nonsurgical therapies do not lead to good outcomes. Previous studies have shown that ventriculoperitoneal shunting, the main treatment option for hydrocephalus, is effective with improved neurological outcomes.^2^ Endoscopic third ventriculostomy has emerged in the last decades as a resultful method to restore CSF flow. Although surgery is the mainstay of hydrocephalus treatment, the rate of complications caused by shunt surgery, such as shunt extrusion, obstruction, or infection, ranges from 17% to 33%.^3^, ^4^, ^5^ The most concerned complication is shunt obstruction.^6^ The rate of shunt revision caused by obstruction and other factors was 32% to 63%.^7^, ^8^, ^9^, ^10^ Besides, the high costs of surgery and medical equipment add heavy medical and social burdens.^11^, ^12^ In the United States, the treatment cost of shunting surgery has reached 1 billion dollars per year,^13^ and the first readmission also generated about 2.25 billion dollars in hospital charges.^7^ It is reasonable to assume that these costs are likely to be higher in developing countries, resulting in heavy household and socio‐economic burdens. Therefore, nonsurgical therapies to reduce the damages and burdens from hydrocephalus are needed as alternatives or adjuvants to ventriculoperitoneal shunting or endoscopic third ventriculostomy.

To unveil the pathogenesis of brain damage in hydrocephalus and develop more effective nonsurgical treatments, researchers have developed different animal models such as intraventricular injection of blood or kaolin to induce hydrocephalus in rats.^14^, ^15^ At the same time, some congenital hydrocephalus models characterized by aqueduct stenosis, ependymal stripping, and astrocytes activation have also been applied.^16^, ^17^ Although animal models cannot fully simulate human body condition, they still play important roles in hydrocephalus research. By using these models, researchers have been looking for possible targets for hydrocephalus treatment. Some targets have been identified, and pharmacological targeted therapies have been used in clinical practice, such as mannitol and furosemide. In addition to drug therapies, other nonsurgical therapies such as mesenchymal stem cells (MSC) transplantation and gene therapy have also shown some success in animal models in recent years.

**FIGURE 1:**
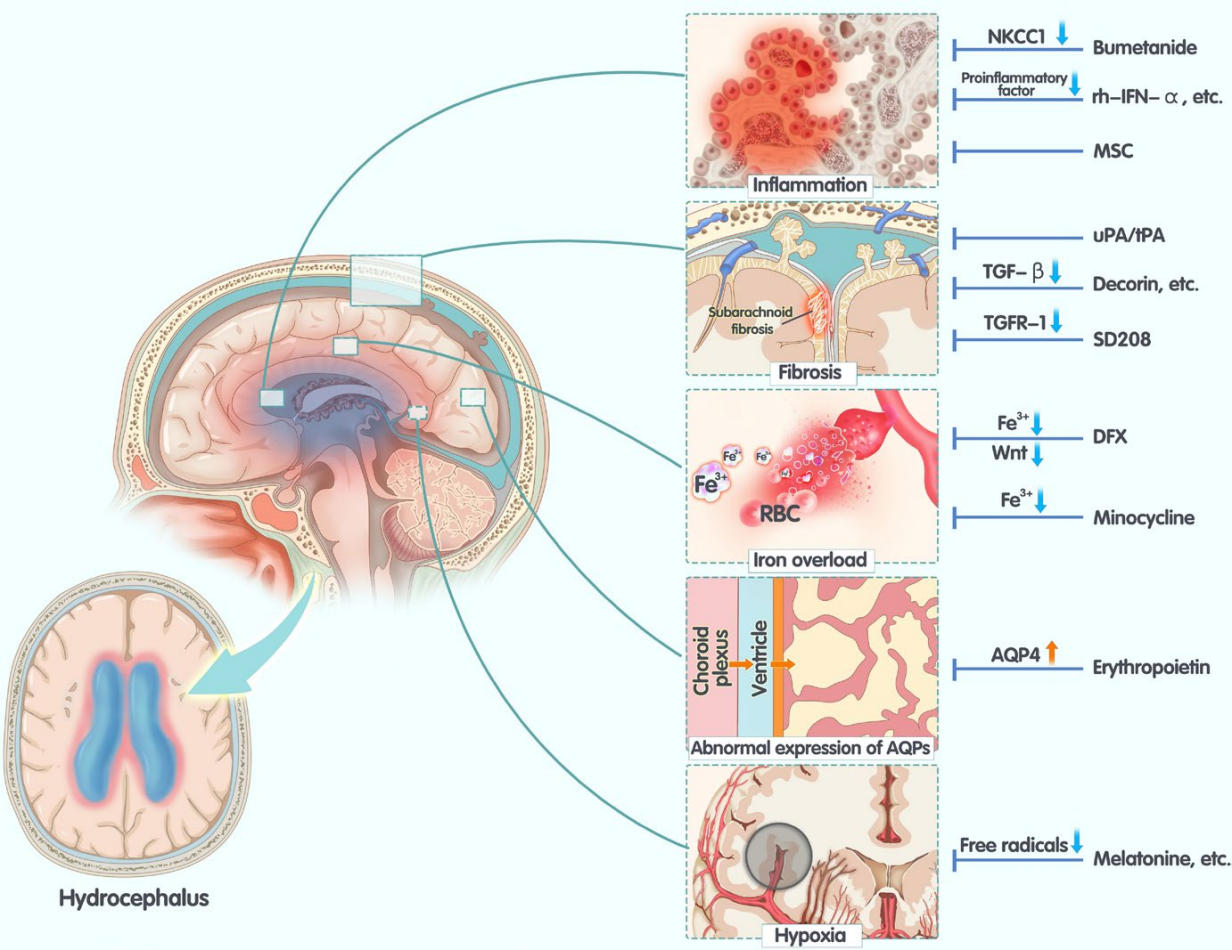
The pathogenesis of hydrocephalus and corresponding treatment Choroid plexus inflammation causes excessive secretion of cerebrospinal fluid. Subarachnoid fibrosis results in obstruction of hydrocele circulation. Iron overload caused by bleeding causes damage to neurons and the blood‐brain barrier, thereby promoting hydrocephalus. Aquaporin‐1 (AQP1) expressed in the choroid plexus is mainly involved in the production of cerebrospinal fluid, while AQP‐4 is expressed mainly in the ependyma, and astrocytes are mainly involved in the absorption of cerebrospinal fluid. Abnormal expression of AQP1 and AQP4 may result in the accumulation of cerebrospinal fluid. Tissue damage caused by oxidative stress may be involved in the development of hydrocephalus. On the right‐hand side of the figure are some of the treatments that correspond to the mechanism. Abbreviations: MSC, mesenchymal stem cell; HGF, hepatocyte growth factor; uPA, urokinase‐type plasminogen activator; TGF, transforming growth factor; sFRP‐1, secreted frizzled‐related protein‐1; LSKL, leucine‐serine‐lysine‐leucine peptide; DFX, desferrioxamine; EPO, erythropoietin; GSH, glutathione

Based on the pathogenesis of hydrocephalus, we have summarized and evaluated the trials on possible targets and studies on nonsurgical therapies for hydrocephalus in animal models (Table 1) and suggested some targeted therapies (Figure 1). The purpose of this review was to summarize the therapeutic effects of different methods and to provide new orientations for animal research and clinical practice for hydrocephalus.

**TABLE 1:** Treatments and their efficacy in animal models of hydrocephalus

Treatment strategies	Models	Histological/biochemical change	Organic/functional change	References
TAK‐242	Rats with IVH	Reduced phosphorylated SPAK and NKCC1	Reduced CSF hypersecretion	19
PDTC	Rats with IVH	Reduced p65 nuclear translocation; reduced abundance of CD68+ cells	Reduced CSF secretion; reduced ventricular dilatation	19
Bumetanide	Rats with IVH		Reduced CSF secretion; reduced ventricular dilatation	19
rh‐IFN‐α	Rats with GMH	Increased phosphorylated JAK1, STAT1, and TRAF3; reduced phosphorylated NF‐κB, IL‐6, and TNF‐α	No significantly improved behavior	26
Mesenchymal stem cell injection	Rats with IVH	Reduced inflammatory cytokines; inhibited astrogliosis	Attenuated compression of the corpus callosum; improved behavioral impairment	32
Mesenchymal stem cells injection	Rat pups with IVH	Reduced inflammatory cytokines; inhibited apoptosis and astrogliosis	Increased corpus callosum thickness; improved behavioral impairment	34
uPA	Rats with hydrocephalus	Reduced deposition of laminin and fibronectin; inhibited gliosis	Reduced ventricular dilatation; improved learning and memory	37
uPA, tPA	Rats with IVH		Reduced ventricular dilatation; no change in hematoma and edema volumes	43
Decorin	Rats with hydrocephalus	Reduced TGF‐β1, phosphorylated Smad2/3; inhibited the deposition of extracellular matrix molecules, laminin, and fibronectin	Reduced ventricular dilatation	52
Decorin	Rats with SAH	Reduced TGF‐β1, p‐Smad2/3, and collagen I	Reduced lateral ventricular index; improved neurocognitive deficits	59
LSKL peptide	Rats with SAH	Reduced TGF‐β1 and p‐Smad2/3	Improved long‐term cognitive deficits	53
Dabigatran	Rats with GMH	Reduced phosphorylated mTOR and p70s6k	Improved long‐term neurofunctional recovery	56
Pirfenidone and losartan	Rat pups		No significant change in ventricular dilatation; improved neurological deficits	62
sFRP‐1	Rats with hydrocephalus	Reduced β‐catenin and cyclin D‐1; inhibited gliosis		71
Minocycline	Rats with GMH	Reduced ferritin; inhibited neuronal death	Reduced ventricular dilatation; improved long‐term motor function	82
Minocycline	Rat pups with GMH	Inhibited activation of microglia	Reduced brain edema and lateral ventricular dilatation; enhanced cortical thickness	84
Erythropoietin	Rat pups with hydrocephalus	Increased aquaporin‐4 in the periventricular ependymal lining and cultured astrocytes; reduced denudation of ependymal line	Reduced ventricular dilatation	122
Melatonin	Rats with hydrocephalus	Increased tissue glutathione; reduced NO		131
Antioxidant mixture	Rat pups with hydrocephalus	No significant change	No significant change in behavior	132

Abbreviations: CSF, cerebrospinal fluid; GMH, germinal matrix hemorrhage; IVH, intraventricular hemorrhage; LSKL, leucine‐serine‐lysine‐leucine peptide; NF‐κB, nuclear factor‐kappaB; NO, nitric oxide; sFRP‐1, secreted frizzled‐related protein‐1; PDTC, ammonium pyrrolidinedithiocarbamate; SAH, subarachnoid hemorrhage; TGF‐β, transforming growth factor‐β; tPA, tissue‐type plasminogen activator; uPA, urokinase‐type plasminogen activator.

## 2 ANTI‐INFLAMMATORY TREATMENT FOR HYDROCEPHALUS

In preterm infants with intraventricular hemorrhage (IVH), fetal ventriculitis, subarachnoid hemorrhage (SAH), and other diseases, inflammation can induce ependymal scarring, intraventricular obstruction, and excessive secretion of CSF by choroid plexus epithelial cells, which can lead to CSF circulation disorder and impaired absorption function.^18^, ^19^ The choroid plexus epithelium (CPe) is the secretory epithelium that can secrete CSF. Adjacent choroid plexus epithelial cells have tight connections and bonding bands near the apex, forming the blood‐brain barrier (BBB). The CPe can function as a barrier that separates the blood and CSF but allow circulating immune cells to enter the brain. Danger‐associated molecular patterns and pathogen‐associated molecular patterns enter the CSF and bind Toll‐like receptor 4 (TLR4) expressed by CPe, thus promoting nuclear translocation of nuclear factor‐κB (NF‐κB) Meanwhile, the synthesis and release of downstream pro‐inflammatory cytokines by active astrocyte or microglia are aroused.^18^, ^20^ These cytokines can bind receptors on the surface of CPe and may induce inflammation and CSF hypersecretion. In this process, some drugs that antagonize TLR4‐NF‐κB signaling or the STE20/SPS1‐related proline/alanine‐rich kinase (SPAK)‐Na+/K+/2Cl− co‐transporter‐1 (NKCC1) complex might be promising to clinical practice. Karimy et al used a recently developed method to directly measure the rate of CSF secretion of the lateral ventricle CPe in live rats. They found that the delivery of TLR4 inhibitor TAK‐242 and NF‐κB inhibitor ammonium pyrrolidinedithiocarbamate can significantly reduce the post‐IVH CSF secretion rate and ventriculomegaly. Similar results have been shown in studies on pharmacological and genetic inhibition of the SPAK‐NKCC1 complex.^19^ Among these results, bumetanide shows its potential to treat hydrocephalus. However, following systemic administration, the intracerebral concentration level of bumetanide is typically lower than the needed concentration to inhibit NKCC1, which critically limits its clinical use for treating brain disorders. In addition to the low permeability of the BBB, active efflux of bumetanide can also explain the extremely low intracerebral concentrations.^21^

Animal research and clinical practice have proven that abnormal expression of pro‐inflammatory and anti‐inflammatory mediators also plays an important role in inflammation induced by hydrocephalus.^22^, ^23^ These inflammatory cytokines may be mainly secreted by astrocytes and microglia. In congenital or acquired neonatal hydrocephalus, astrocyte‐ and microglia‐mediated neuroinflammation seems to be involved in the development of hydrocephalus.^16^ For example, GFAP and Iba‐1 immunoreactivity increased in the parietal cortex of rats with hydrocephalus induced by injection of kaolin on postnatal day 1, indicating significant activation of astrocytes and microglia.^24^ Analysis of the gene expression data also showed an increase in neuroinflammation.^24^ During this process, astrocytes may be stimulated by various pro‐inflammatory factors secreted by activated microglia, such as IL‐1, and thus become activated, hence aggravating neuroinflammation.^25^ Meanwhile, in addition to neuroinflammation, the activation and proliferation of glial cells are also involved in other pathophysiological processes of hydrocephalus, such as the regulation of aquaporin‐4 (AQP4) expression and oxidative stress. Some drugs that target glial cells may be an option for hydrocephalus treatment.

In a study on the germinal matrix hemorrhage (GMH) model, rh‐IFN‐α effectively controlled post‐hemorrhage hydrocephalus (PHH) by inhibiting microglial activation through JAK1‐STAT1/TRAF3/NF‐κB signaling and reducing the secretion of pro‐inflammatory cytokines.^26^ However, a traditional anti‐inflammatory agent showed that treatment could not significantly inhibit the progression of hydrocephalus in both animal experiments and clinical studies of dexamethasone.^27^, ^28^, ^29^ Therefore, inhibition of the expression and secretion of pro‐inflammatory factors may be a feasible way to relieve hydrocephalus. But not all drugs that inhibit the expression of inflammatory cytokines are effective.

Aside from drugs, MSC has shown anti‐apoptotic, anti‐inflammatory, antifibrotic, and antioxidative paracrine potential in the treatment of many neurological diseases.^30^, ^31^ In a study, rat pups with severe IVH were injected with MSC‐buffered saline into the right ventricle. The results showed that transplanting MSC significantly attenuated PHH following severe IVH. This might be due to the anti‐inflammatory effect of MSC.^32^, ^33^ Another study also showed that MSC can downregulate the expression of inflammatory cytokines, such as IL‐1α, IL‐1β, and IL‐6, and inhibit overactive astrocytes. As the therapeutic time window in the study was limited to the early phase of inflammation following severe IVH,^34^ more studies are required to verify its effects. And for successful clinical translation of MSC transplantation, the optimal route, timing, dosage, and short‐ and long‐term safety are critical issues that remain to be addressed.

## 3 ANTIFIBROTIC TREATMENT FOR HYDROCEPHALUS

Fibrosis is the formation of excessive connective tissue following the repair of inflammation. Too much fibrillar connective tissue formation may disrupt the normal function of surrounding tissue. Subarachnoid fibrosis secondary to cerebral hemorrhage is an important mechanism in the pathophysiology of chronic hydrocephalus affecting the normal circulation and absorption of CSF.^35^ Extensive fibrosis, mainly caused by excessive extracellular matrix (ECM) production, in subarachnoid space, may play an important role in the development of PHH and other forms of communicating hydrocephalus.^36^ Excessive ECM deposition obstructs CSF flow and reduces CSF reabsorption. Injecting kaolin into the basal cistern can cause excessive deposition of fibronectin and laminin, which are two main components of ECM.^37^ Therefore, targeting subarachnoid fibrosis may be helpful in the treatment of hydrocephalus.

The urokinase plasminogen activator (uPA) is a serine protease that converts plasminogen to plasmin.^38^ Plasmin is a protease that can degrade fibrin and ECM components.^39^ uPA may relieve fibrosis and ECM deposition in the subarachnoid space by inhibiting the deposition of laminin, fibronectin, and extracellular matrix molecules in rats injected with kaolin, thus inhibiting the development of hydrocephalus.^37^, ^40^ The release and activation of hepatocyte growth factor promoted by uPA may also play an important role in this process.^41^ Besides the antifibrotic effects, uPA treatment of PHH has a mechanical benefit of accelerating the clearance of blood clots and improving the blocked CSF reflux pathway caused by clotting, thus facilitating CSF reflux. Tissue plasminogen activator (tPA) is a kind of plasminogen activator that belongs to the same serine protease family as uPA.^42^ Gaberel et al compared the effects of these two drugs by infusing them separately into the ventricle of rats with hematoma and IVH induced by injection of collagenase type VII near the ventricle wall. They found that although both uPA and tPA can reduce ventricular dilatation caused by post‐IVH hydrocephalus, only uPA significantly improved functional recovery.^43^ It possibly results from the potential pro‐inflammatory and toxic effects of tPA.^44^, ^45^ However, the use of uPA has been associated with an increased risk of intracranial rebleeding and infection in clinical studies of IVH,^46^, ^47^ which may limit its clinical use.

Transforming growth factor‐β (TGF‐β) plays an important role in promoting fibrosis and inflammation. It can be activated by many factors and released from activated microglia or platelet.^48^, ^49^ TGF‐β stimulates mesenchymal stem cells and fibroblasts to produce ECM matrix proteins, which may disrupt CSF flow.^50^ In clinical practice, the occurrence of PHH in premature infants is associated with the increase in TGF‐β1 and ECM protein expression in CSF.^51^ Therefore, TGF‐β may be a vital target in hydrocephalus treatment. In particular, the TGF‐β1/Smad pathway may play an important role in the development of hydrocephalus. In the kaolin‐induced hydrocephalus model of rats, TGF‐β/Smad2/3‐mediated subarachnoid fibrosis and development of hydrocephalus were inhibited by sustained intraventricular decorin infusion.^52^ And in another rat model of SAH, the leucine‐serine‐lysine‐leucine peptide, a little peptide of four amino acids, has also been used to prevent the development of chronic hydrocephalus by reducing the activation of latent TGF‐β1/Smad2/3 signaling pathway following SAH.^53^ In addition, TGF‐β is associated with injury induced by thrombin in SAH models.^48^, ^54^ Thrombin is also one of the important factors causing PHH.^55^ It is reported that dabigatran, a thrombin antagonist, significantly diminished post‐hemorrhagic ventricular dilation and white matter loss.^56^ The activation of protease‐activated receptors‐1 plays an important role in damage caused by thrombin.^56^, ^57^, ^58^

Researchers tried to interfere with the TGF‐β pathway as a means to treat hydrocephalus in animal models.^59^, ^60^ Manaenko et al administered SD208, a TGF receptor I inhibitor, daily for 3 days after GMH induction and compared the result with vehicle‐treated GMH rats. The results showed that high‐dose SD208 inhibited GMH‐induced activation of the TGF‐β pathway, reduced the deposition of vitronectin, and alleviated brain atrophy and hydrocephalus.^61^

However, some other drugs that inhibit TGF‐β may not affect hydrocephalus. Pirfenidone and losartan reduce TGF‐β expression and have antifibrotic potential in other organs. They were used to treat seven‐day‐old rats with post‐IVH hydrocephalus, but neither drug had a beneficial effect on ventricle size or behavior after treatment.^62^ Although treatment with pioglitazone reduced glial activation in mice with overexpressing TGF‐β1, it promoted hydrocephalus unexpectedly.^63^ The mechanism of this interesting phenomenon remains to be investigated through more experiments.

## 4 ANTI‐IRON OVERLOAD TREATMENT FOR HYDROCEPHALUS

After IVH or SAH, hemoglobin (Hb), iron, and other substances in blood may be released into CSF and accumulate in the brain parenchyma and CSF in subarachnoid space, leading to brain injury and ventricular dilation.^55^, ^57^, ^64^ Molecules related to inflammation, such as TNF‐α, monocyte chemotactic protein‐1, IL‐1β, IL‐6, and IL‐8, are highly expressed in rat pups that received intraventricular injection of Hb.^65^, ^66^ Hb is broken down into heme, and heme is broken down by heme oxygenase (HO) into iron, carbon monoxide, and biliverdin.^55^ Strahle et al injected protoporphyrin IX, the iron‐deficient immediate heme precursor into the ventricle. It did not result in ventricular enlargement, while both Hb and iron injection could lead to a continuous increase in ventricular size.^67^ Although Hb and its degradation products are likely to contribute to ventricular enlargement in different ways, iron may play a major role in this process by inducing ependymal cell death and cilia loss.^68^ It has been observed that the levels of hemoglobin and ferritin in CSF of neonates with PHH or IVH were positively correlated with the size of the ventricle.^69^ But there was no significant change in the level of iron scavenging proteins.^69^ These results suggest that disturbance of iron clearance may be involved in the pathogenesis of PHH. Besides, it has been reported that deferoxamine, a kind of iron chelator, could inhibit Wnt1 and Wnt3a gene expression and protein synthesis in IVH‐induced hydrocephalus rats, in addition to its own iron chelation function.^70^ This suggests that iron may be a key stimulant that activates the Wnt signaling pathway. Some studies have shown that the Wnt signaling pathway may be associated with inflammation, fibrosis, reactive gliosis, coagulation cascade, and lipid peroxidation in the development of hydrocephalus.^55^, ^71^, ^72^, ^73^ Disturbed Wnt signal due to CCDC88C mutation also leads to an autosomal recessive nonsyndromic hydrocephalus.^74^

It is worth noting that the breakdown products of heme in addition to iron, including HO‐1 itself, are possibly involved in the regulation of brain damage.^75^, ^76^ Studies have shown that using HO inhibitors such as protoporphyrin IX and zinc protoporphyrin could reduce intracerebral hemorrhage‐induced inflammation, brain atrophy, and the size of the hematoma and edema.^77^, ^78^ But Zhang et al suggested that the role of HO‐1 activation in experimental cerebral hemorrhage may be bimodal. It mediates brain damage early after hemorrhage but promotes nervous system recovery later in the course of disease.^79^ HO‐1 exerts anti‐inflammatory and antioxidant effects not only through bilirubin and carbonic oxide, and its catalytic products, but also through inhibiting the expression of inflammatory factors and blocking NF‐κB.^75^, ^80^ Therefore, it is very important to determine the regulatory mode of HO and the eventual time window for treatment.

Minocycline is another promising treatment for intracranial hemorrhage due to its ability to chelate iron.^81^ Guo et al confirmed that minocycline could reduce iron accumulation in an experimental GMH‐IVH model, thus reducing the risk of brain damage and hydrocephalus.^82^ At the same time, as a widely used anti‐inflammatory drug, minocycline is also a macrophage/microglia inhibitor by various pathways such as poly (ADP‐ribose) polymerase‐1 signaling pathway or by activating the cannabinoid receptor 2.^83^, ^84^ Other evidence suggests that administration of minocycline may reduce hydrocephalus by inhibiting reactive gliosis in animal models with congenital or acquired hydrocephalus.^54^, ^85^, ^86^ Minocycline also showed a therapeutic effect of delaying hydrocephalus in spontaneously hypertensive rats.^87^ Progressive ventricular dilation in spontaneously hypertensive rats has been reported in 1986.^88^ The ventricles of these rats remained enlarged, even after the blood pressure was lowered by captopril.^89^ Current studies have found that choroid plexus cell death, ependymal injury, impaired glymphatic transport, hemorrhage, and other factors may be involved in the development of hydrocephalus in spontaneously hypertensive rats.^17^, ^87^, ^90^, ^91^ Therefore, minocycline is a promising drug for the treatment of hydrocephalus and related tissue damage. However, minocycline has some side effects, such as the possibility of inducing lupus erythematosus.^92^

## 5 AQUAPORIN REGULATION TREATMENT FOR HYDROCEPHALUS

AQPs are transmembrane functional units distributed throughout the body that allow water molecules or other small molecules to pass through.^93^ They are involved in other physiological or pathological processes beyond water homeostasis, such as cell‐cell adhesion, facilitating gas and cation transportation, inflammation, etc.^94^, ^95^ In the brain, AQPs are related to a variety of pathological processes, including cerebral ischemia/reperfusion injury, brain edema, and hydrocephalus.^96^, ^97^ AQP1 and AQP4 are the main kinds of AQPs related to hydrocephalus.^98^, ^99^

AQP1 is mainly expressed on the apical and basolateral surfaces of the choroid plexuses and plays a role in CSF production.^100^ Acetazolamide may reduce CSF production by inhibiting AQP1 and thus slow the development of hydrocephalus. However, a study in six dogs with internal hydrocephalus showed that acetazolamide treatment was not effective in inhibiting ventricular dilation.^101^ Despite the fact that many general practitioners and neurologists are still prescribing acetazolamide, acetazolamide is not recommended to treat hydrocephalus in clinic.^102^, ^103^ In addition, there have been experiments using gene therapy to regulate AQP1 expression to restore glandular function.^104^, ^105^ Although gene therapy for hydrocephalus has not yet been tested in animal models, it does provide a direction for relevant experiments.

AQP4 is expressed in ependymal and glial cells.^106^ Compared with wild‐type mice, AQP4‐null mice with obstructive hydrocephalus are observed with significantly increased CSF content and accelerated ventricular enlargement progression, suggesting that AQP4 may be involved in CSF reabsorption.^107^ At the same time, enhanced AQP4 immune response was observed in rat brains with severe hydrocephalus.^108^ These results suggest that the upregulation of AQP4 expression may be a compensatory response to maintain hydrocephalus homeostasis.

The glymphatic system is a sleep‐assisted CSF and interstitial fluid transport system that promotes the removal of waste from the brain parenchyma.^109^, ^110^ In this pathway, fluid and solutes enter the perivascular space of the artery and then diffuse into the brain parenchyma by AQP4 expressed on the endfeet of astrocytes that ensheathe the brain vasculature.^111^, ^112^ These substances into the brain parenchyma are collected in the perivenous spaces surrounding the large deep veins and flow into the neck lymphatic system.^109^ Some evidence suggests that there may be impaired glymphatic transport in patients with idiopathic normal pressure hydrocephalus (iNPH) and that the abnormal expression of AQP4 may be involved in this damage.^91^, ^113^, ^114^, ^115^ By using magnetic resonance imaging contrast agent as CSF tracer, it was found that there is a significant delay in the tracer clearance stage of iNPH patients.^115^, ^116^ This delayed clearance may be related to the deposition of amyloid‐β peptides (Aβ) in the brain tissues of iNPH patients, but the mechanism remains to be explored. A study showed that the expression of AQP4 and its anchor molecule dystrophin‐71 in the perivascular endfeet of astrocytes decreased in iNPH patients.^117^ Thus, in iNPH, the reduction of AQP4 in the perivascular endfeet may impede the transport of fluid and solutes along the microvessels. Evidence shows that deletion of AQP4 does not alter Aβ levels in the brain of adult mice,^118^ but does decrease exogenous Aβ clearance,^119^ suggesting that AQP4 may play an important role in the development of Aβ‐related diseases. More experiments are needed to investigate the connection between the clearance delay and the accumulation of Aβ and the role of AQP4.

Erythropoietin (EPO), a multifunctional molecule that has anti‐inflammatory and angiogenesis function to reduce brain injury,^120^, ^121^ can also influence the expression of AQP4. Siddiqui et al injected EPO intraperitoneally in kaolin‐induced hydrocephalus rats for five consecutive days. The result showed that EPO treatment significantly reduced the expression of miR‐130a and increased the expression of miR‐668, thereby upregulating the expression of AQP4 in cultured ventricular septal epithelial cells and astrocytes.^122^ miR‐668 may be a potent expression activator of AQP4 in response to EPO, but there may be many other activators. Therefore, targeting AQP4 may be a promising direction for the treatment of hydrocephalus.

## 6 ANTIOXIDATIVE STRESS TREATMENT FOR HYDROCEPHALUS

Many clinical and experimental studies have shown that chronic hydrocephalus is related to decreased cerebral blood flow and oxygen delivery to the brain.^123^ It has been shown that chronic hypoxia could cause older rats to exhibit the characteristics of adult chronic hydrocephalus, including increased ventricular size, slightly increased intracranial pressure, and cognitive deficits in the brain.^124^ Besides, free radicals, such as reactive oxygen species and reactive nitrogen species, are also associated with oxidative damage to neurons and other brain cells.^125^, ^126^ Oxidative stress induced by hypoxia/ischemia environment involving lipid peroxidation and oxidative and nitrosylative reaction may be one of the mechanisms of brain injury in hydrocephalus. These reactions may be ongoing even while ventricle expansion stops.^127^

It should be noticed that nitric oxide (NO) plays a role in oxidative stress as well. Increasing citrulline and nitrate concentration, markers of NO production, were found in CSF of patients with acute hydrocephalus, indicating more NO production.^128^ NO can expand blood vessels and relieve ischemia and hypoxia, but may also damage the BBB. NO mediates the opening of the BBB, allowing metabolic waste products in blood to enter the brain. The most harmful effect of NO is that it can be oxidized to peroxynitrite, which can cause wide cellular damage by oxidizing proteins, DNA, etc.^129^, ^130^

Antioxidants might be a possible treatment for damage followed by oxidative stress in hydrocephalus. Melatonin, a hormone secreted by the pineal gland, has shown its protective effect by scavenging free radicals. Rats with hydrocephalus induced by kaolin injection received melatonin treatment, and the result showed that melatonin could stop the elevations in NO levels of choroid plexus tissue and delay the decrease in glutathione, which is also a free radical scavenger and antioxidants.^131^ It shows the potential to inhibit inducible NO synthase activity to reduce NO production.

However, some studies reported that some antioxidant therapies had no benefits in rats with hydrocephalus. Rats received an injection of kaolin into the cisterna magna to induce hydrocephalus. Then, they were treated for two weeks daily with a low or high dose of an antioxidant mixture containing α‐tocopherol, L‐ascorbic acid, coenzyme Q10, reduced glutathione, and reduced lipoic acid. It is interesting that although all agents used in the study affected oxidative stress, all groups developed significant ventricle expansion and exhibited white matter damage.^132^ A possible explanation is that oral administration is not as effective as intraperitoneal injection. In a study on quercetin, an antioxidant widely found in fruits and vegetables, the results showed no significant benefit on kaolin‐induced hydrocephalus in rats even though quercetin has shown therapeutic effect in animal models of other nervous system damage.^133^

Angiogenesis is a protective mechanism under the condition of ischemia and hypoxia.^134^ It is reported that vascular endothelial growth factor (VEGF), which has a mitogenic activity and increases the vascular permeability effect on endothelial cells, is highly expressed in the CSF of premature infants with PHH or adult chronic hydrocephalus patients.^135^ In rats that were under chronic hypoxia, hypoxia‐inducible factor‐1alpha rapidly accumulated and enhanced the expression of VEGF.^136^ The use of bevacizumab, an anti‐VEGF antibody, can relieve the symptoms caused by VEGF injection, but more research should be done to investigate the effect of VEGF on hydrocephalus.^137^ However, we should be aware of the destructive force of VEGF, which may outweigh its angiogenic force in hydrocephalus.^138^ It is reported that injection of VEGF leads to ventriculomegaly, denudation of ependymal cells, and loss of cilia in rats. Therefore, the regulation of VEGF and its receptors should be taken into account as considered as a treatment option for hydrocephalus.

## 7 CONCLUSIONS AND PERSPECTIVE

Animal models with hydrocephalus, including congenital or acquired hydrocephalus, can simulate pathogenesis and pathological characteristics of human hydrocephalus to some extent. Exploring the pathophysiology and new therapeutic targets of hydrocephalus through these animal models will help scientists to explore the feasibility and effectiveness of nonsurgical treatments.

Inflammatory targeting therapy is helpful in inhibiting the development of hemorrhagic hydrocephalus and post‐infection hydrocephalus, and its main mechanisms are associated with acute CSF oversecretion and scarring of the CSF drainage pathway.^18^ Modulating the expression of certain molecules in the TLR4‐NF‐κB signaling pathway or the SPAK‐NKCC1 co‐transporter complex may relieve neuronal damage caused by inflammation in hydrocephalus. Considering suppressing only one or a few factors may not yield a significant therapeutic effect, many other experiments need to be conducted to explore the optimal performance.

Targeting subarachnoid fibrosis is another promising treatment for hydrocephalus. Among all the experimental results we have referred to, uPA shows its potential in the alleviation of fibrosis and ECM deposition, if its side effects of rebleeding and infection can be minimized. Besides, interfering with TGF‐β and its pathway also seems to be effective. Considering some drugs that block TGF‐β show no differences in the management of hydrocephalus, it is suggested that more similar experiments should be conducted to exclude the ineffective therapies.

For the reason that heme degradation and iron deposition are important culprits of hydrocephalus if they are released in the CSF and impair CSF absorption, more and more attention has been paid to anti‐iron overload treatment for hydrocephalus Through animal experiments, researchers have found that deferoxamine, a kind of iron chelator, can inhibit Wnt1 and Wnt3a gene expression and protein synthesis, which might contribute to the management of hydrocephalus. The same result has also been demonstrated by minocycline, which has the ability to chelate iron. The positive outcome in animal models mentioned above suggests that targeting iron overload is likely to be a sensible way to treat hydrocephalus.

As for the function of AQPs, more experiments are now needed to demonstrate the regulatory role of AQPs in the pathogenesis of hydrocephalus and possible treatment approaches that target AQPs. The glymphatic system theory may give new impetus to the study of the mechanism and treatment of hydrocephalus.

Considering oxidative stress induced by hypoxia or ischemia environment may be one of the mechanisms of brain injury in hydrocephalus, we have discussed the role of antioxidants and the regulation of VEGF in animal models with hydrocephalus and found that melatonin might offer benefits by scavenging free radicals and that the use of anti‐VEGF antibody may relieve the symptoms caused by the injection of VEGF. These experimental results may give inspiration for researchers to pursue the possible treatments for hydrocephalus concerning antioxidative stress.

Although there are few studies on stem cell and gene therapy for hydrocephalus, these emerging therapies are potential directions that are worth investigating deeply.

In a word, nonsurgical treatment for hydrocephalus in animal models has been studied for many years, but no significant results have been obtained at the clinical translation stage. As our understanding of the pathogenesis of hydrocephalus improves and more treatment options are validated, we hope to make great strides in the nonsurgical treatment of hydrocephalus.

## CONFLICT OF INTEREST

The authors declare that they have no competing interests.

## Data Availability

The datasets used or analyzed during the current study are available from the corresponding author (GLX) on reasonable request.
